# Determinants of women’s preferred and actual abortion provision locations in Nigeria

**DOI:** 10.1186/s12978-021-01290-w

**Published:** 2021-11-27

**Authors:** Meagan E. Byrne, Elizabeth Omoluabi, Funmilola M. OlaOlorun, Caroline Moreau, Suzanne O. Bell

**Affiliations:** 1 Department of Population, Family and Reproductive Health, Johns Hopkins University Bloomberg School of Public Health, Baltimore, MD USA; 2 Centre for Research, Evaluation Resources and Development, Ile-Ife, Nigeria; 3 Statistics and Population Studies Department, University of the Western Cape, Cape Town, South Africa; 4 Department of Community Medicine, University of Ibadan, Ibadan, Nigeria; 5 Soins et Santé Primaire, CESP Centre for Research in Epidemiology and Population Health U1018, Inserm, 94805 Villejuif, France

**Keywords:** Abortion, Decision-making, Nigeria

## Abstract

**Background:**

Unsafe abortion remains a leading cause of maternal mortality globally. Many factors can influence women’s decisions around where to seek abortion care; however, little research has been done on abortion care decisions at a population-level in low-resource settings, particularly where abortion is legally restricted.

**Methods:**

This analysis uses data from a 2019–2020 follow-up survey of 1144 women in six Nigerian states who reported an abortion experience in a 2018 cross-sectional survey. We describe women’s preferred and actual primary abortion care provider/location by distinguishing clinical, pharmacy/chemist, or other non-clinical providers or locations. We also examine factors that influence women’s decisions about where to terminate their pregnancy and identify factors hindering women’s ability to operationalize their preferences. We then examine the characteristics of women who were not able to use their preferred provider/location.

**Results:**

Non-clinical providers (55.0%) were more often used than clinical providers (45.0%); however, clinical providers were preferred by most women (55.6%). The largest discrepancies in actual versus preferred abortion provider/location were private hospitals (7.6% actual versus 37.2% preferred), government hospitals (4.3% versus 22.6%), chemists (26.5% versus 5.9%) and pharmacies (14.9% versus 6.6%). “Privacy/confidentiality” was the most common main reason driving women’s abortion provider/location choice (20.7%), followed by “convenience” (16.9%) and “recommended” by someone (12.3%), most often a friend (60.8%), although top reasons differed by type of provider/location. Cost and distance were the two most common reasons that women did not use their preferred provider/location (46.1% and 21.9%, respectively). There were no statistically significant differences in the sociodemographic characteristics between women who were able to use their preferred provider/location and those who were not able to implement their preferred choice, with the exception of state of residence.

**Conclusions:**

These findings provide insights on barriers to abortion care in Nigeria, suggesting discretion is key to many women’s choice of abortion location, while cost and distance prevent many from seeking their preferred care provider/location. Results also highlight the diversity of women’s abortion care preferences in a legally restrictive environment.

## Plain language summary

Many factors influence a woman’s pathway to obtaining an abortion, even in a setting with strict laws prohibiting the practice. This study aims to explore where women in Nigeria would prefer to and actually obtain their abortions, reasons why they could or could not use their preferred provider/location, and differences between women who were and were not able to use their preferred provider/location.

The findings show that most women would opt to use a clinical source, such as a government or private hospital, especially among women who did not use their preferred source. Privacy/confidentiality, convenience, and recommendation from someone like a friend or partner drove women’s abortion care preferences, although these influences differed by type of provider/location (clinical, pharmacy/chemist, or other non-clinical). Issues like cost, distance, and lack of privacy were barriers that prevented women from using their preferred provider/location, instead obtaining their abortion from a less desired provider. However, the sociodemographic characteristics of women who did and did not use their preferred provider/location did not differ significantly, except by state.

These findings provide insights on barriers to safe abortion care in Nigeria, suggesting social safety drives many women to seek care outside of the healthcare system, while cost and distance prevented many from seeking clinical services. Results also highlight the diversity of women’s abortion preferences in a setting where abortion is legally restricted.

## Introduction

Unsafe abortion remains a leading cause of maternal mortality, contributing to 8–15% of maternal deaths globally [1, 2]. While abortion rates do not vary widely by geographic setting, abortion safety is greatly impacted by the legal environment [3]. Legal restrictions not only inhibit access to safe methods and skilled care but may also delay care-seeking and impede access to accurate information and post abortion care (PAC) for treatment of complications [4]. Most abortions worldwide take place in legally restrictive settings, thus despite the high safety profile of abortion when done in accordance with medical standards [5], unsafe abortion remains a significant threat to maternal health.

In recent years, abortion safety in legally restrictive settings has improved with increased access to safe, effective abortion medications like Misoprostol at the community level [6]. As safe abortion methods become more accessible, exploring the broader aspects of quality of abortion care is necessary to improve service delivery and reduce disparities in abortion outcomes. Even in legally restrictive settings, women may be able to exercise a degree of choice in determining their abortion care and many factors can influence women’s decisions around where to seek services; however, little research has been done on abortion care decision-making at a population level in low-resource settings, particularly where abortion is legally restricted.

In Nigeria, induced abortion is legal only to save the woman’s life, and in Lagos state to preserve the woman’s life and physical health [7, 8]; nonetheless it is a common reproductive health experience. Recent estimates using indirect confidante methodology indicate the one-year abortion incidence was approximately 46 per 1000 women aged 15 to 49 years in 2017 [9]. With unmet contraceptive need at 19% among married women of reproductive age and 48% among unmarried sexually active women [10], women who find themselves pregnant at a time when they do not desire a child may seek out abortion services despite legal restrictions. Nearly two-thirds of abortions are considered most unsafe, involving non-recommended methods (i.e., methods other than surgery or medication abortion drugs) from non-clinical providers [11]. Unsafe abortion contributes to Nigeria’s high maternal mortality ratio, estimated at 512 maternal deaths per 100,000 live births [10]. Better understanding women’s abortion care-seeking and associated decision-making in this setting can inform harm reduction efforts that seek to reduce morbidity and mortality due to unsafe abortion.

In Nigeria, safe abortion options are available, largely a result of increased availability of medication abortion drugs (Mifepristone and Misoprostol) in recent years. Mifepristone was registered for use in 2017 and Misoprostol has been on the country’s Essential Medicines List for incomplete and spontaneous abortion since 2010 [12, 13]. While there is limited data on the use of medication abortion drugs in Nigeria, a recent review of referral hospital medical record data showed an increase in Misoprostol use for induced abortions over a nine-year study period, and this was associated with a reduction in severe morbidity [14]. Women increasingly have an option to self-manage their abortion by purchasing the drugs through pharmacies, chemist shops, or formal harm reduction efforts—like abortion hotlines and accompaniment models—that support women in safely inducing using medication abortion drugs [15, 16]. For facility-based care, evidence suggests that provision of abortion in facilities is not uncommon, with 38% of women reporting use of a private or public facility to terminate their pregnancy in a recent study [11]. Thus, it is clear that some women are better able to navigate barriers to safe abortion methods in this legally restricted setting.

Women’s decisions regarding where to seek abortion care are influenced by a number of factors. Coast et al. developed a comprehensive framework that encompasses the full range of factors that shape women’s abortion care-seeking trajectories [17], which includes the abortion-specific context, women’s individual context, and the international or sub-national context, which have thus far primarily been examined qualitatively or among PAC patients [18–20]. Studies have found that perceived or actual illegality of abortion and poor provider attitudes deterred many women from seeking abortions in formal healthcare settings [21]. Awareness of and trust in abortion care options are also informed by women’s social networks [21, 22]. Financial considerations are a significant factor in women’s abortion care decision-making [20, 22, 23]. Level of discretion afforded by a given provider also factors prominently due to the stigma of abortion even in legally enabling environments [22, 24]. Because much of this research examines the experiences of PAC patients within the formal healthcare system or among smaller qualitative samples, we lack a broader perspective on the factors that influence non-clinical abortion care decisions for women who do not present for care at a formal facility for a legal abortion or PAC and the reasons driving these decisions.

To address this knowledge gap, our study explores one facet of abortion care-seeking trajectories among Nigerian women who terminated a pregnancy in the formal or informal sector. The primary aim of this study is to examine the factors that influenced women’s decisions about where to terminate their pregnancy and their corresponding preferences regarding abortion provider/location. The secondary aim is to examine the reasons that hindered women’s ability to operationalize their preference and to compare characteristics of women who were able and unable to use their preferred abortion provider.

## Methods

### Study design

This analysis uses data from two quantitative surveys conducted in 2018 and 2019–2020 in Nigeria by Performance Monitoring for Action (PMA), which is implemented by the Centre for Research, Evaluation Resources and Development (CRERD) with technical guidance provided by the Gates Institute for Population and Reproductive Health at the Johns Hopkins Bloomberg School of Public Health. PMA currently conducts nationally or regionally representative surveys on family planning and reproductive health in nine countries in Africa and Asia. The 2018 PMA survey in Nigeria, conducted in April and May 2018, was a cross-sectional survey of households and eligible women aged 15–49 years in seven states (Anambra, Kaduna, Kano, Lagos, Nasarawa, Rivers, and Taraba). Details about the 2018 PMA Nigeria survey methodology have been published elsewhere [9]. Women who reported having done something to remove a pregnancy or bring back a period when they were worried they were pregnant in the 2018 survey were asked a series of questions about that experience and if they could be re-contacted for a follow-up survey.

The follow-up survey, which collected more detailed information on the woman’s previously reported abortion experience, was conducted in December 2019 through February 2020 with consented respondents in six of the seven states from baseline (there were very few abortions reported in Kano, thus we chose to exclude these respondents for practical considerations). A total of 1476 women were eligible and willing to be contacted for the follow-up survey and 1144 completed it (response rate: 77.5%). If the woman provided a follow-up phone number at baseline, study staff attempted to contact her by phone to remind her about her participation in the previous PMA survey round and willingness to be re-contacted. If the woman was interested in participating, the staff arranged an interview date, time and location of the woman’s choosing. For women who did not provide a phone number or in cases where study personnel were unable to reach the woman at the number provided, PMA resident interviewers returned to the woman’s community from the previous round to describe the study and ask about participation in person.

All respondents provided verbal informed consent before both the baseline and follow-up surveys to participate. Interviews were conducted face-to-face by PMA interviewers in primarily Hausa, Igbo, Pidgin, Yoruba, or English. Surveys were professionally translated into Hausa, Igbo, Pidgin, and Yoruba from English and piloted with native speakers before data collection. Surveys were programmed into smart phones using OpenDataKit software. Ethical approval was obtained from the Johns Hopkins Bloomberg School of Public Health Institutional Review Board and the National Health Research Ethics Committee of Nigeria.

### Measures

The baseline survey allowed respondents to report either a pregnancy removal or period regulation and the follow-up survey asked about the experience using the language previously reported by the respondent. We refer to both experiences (removing a pregnancy and bringing back a period) as abortions in this analysis. Women could report up to two abortion methods and providers/locations (first and last) if they reported doing multiple things to end the pregnancy. Follow-up survey questions focused on the woman’s experiences using her first (if multiple) or only (if one) method and provider/location; the measures in this analysis focus on the woman’s first/only provider/location and the associated decision-making.

In this analysis, we classify abortion providers/locations as clinical or non-clinical. Clinical provider/locations include government hospitals, government health centers, family planning clinics, mobile clinics (public), private hospitals, private doctors, and mobile clinics (private). Non-clinical provider/locations include pharmacies, chemists (drug shops not operated by pharmacists), public events, fieldworkers (private), shops, friends/relatives, healers, markets, and other. Missing values for first/only provider/location responses (n = 13) were classified as other.

We examined several respondent sociodemographic characteristics in this analysis, including a number of the woman’s characteristics at the time of her abortion, specifically age (categorical), marital status, school attendance, whether she had any children, and rural/urban residence. We were unable to collect information on women’s wealth and state of residence at the time of abortion; therefore, we rely on these measures at the time of women’s baseline survey.

### Analysis

We first used descriptive statistics to examine the sociodemographic characteristics, preferred and actual first/only abortion provider/location, and reasons for using the first/only provider/location among all women in the sample. We also examined the reasons for not using a preferred first/only provider/location among the subset of women who reported that they did not use their preferred provider/location. We explored differences in top decision-making factors by category of first/only provider/location and differences in sociodemographic characteristics between women who did and did not use their preferred provider/location, assessed via design-based F-statistic. The design weights represent the inverse of the probability of selection multiplied by the inverse of the cluster response rate. We multiplied these weights with the loss to follow-up weights, which we calculated by regressing the sociodemographic characteristics of women who reported an abortion in the baseline survey on their odds of completing the follow-up survey and taking the inverse of the predicted probability from that model. All analyses were conducted using Stata 15.1 (College Station, TX).

## Results

The sociodemographic characteristics of follow-up interview participants at the time of their abortion experience are presented in Table 1. At the time of their abortion, most women were under the age of 30 years and at peak childbearing: 2.8% were under 15 years, 18.3% were 15–19 years, 29.2% were 20–24 years, and 22.0% were 25–29 years. Over half of women were married (53.0%), 28.8% were attending school, 48.6% had children, and 69.0% were living in an urban setting.

**Table 1:** Background characteristics of respondents at time of abortion (N = 1144)*

	n (%)
*Age*	
< 15	34 (2.8)
15–19	215 (18.3)
20–24	313 (29.2)
25–29	242 (22.0)
30–34	176 (15.1)
35–39	95 (7.3)
40–44	42 (3.1)
45–49	9 (0.7)
No response	18 (1.6)
*Married/cohabiting*	
No	449 (47.0)
Yes	692 (53.0)
*Was attending school*	
No	855 (71.2)
Yes	288 (28.8)
*Had children*	
No	509 (51.4)
Yes	634 (48.6)
*Wealth^*	
Poorest	170 (12.0)
Second poorest	260 (18.5)
Middle	212 (18.7)
Second wealthiest	261 (25.4)
Wealthiest	237 (25.5)
*Residence*	
Rural	446 (31.0)
Urban	698 (69.0)
*State^*	
Anambra	191 (16.3)
Kaduna	214 (6.0)
Lagos	238 (27.4)
Nasarawa	165 (12.3)
Rivers	273 (28.9)
Taraba	63 (9.1)
Total	1144 (100.0)

*Ns across categories of a characteristic that do not sum to total N is a result of missingness

^From time of baseline survey, not time of abortion

Women were asked for both their actual and preferred first/only provider/location for their abortion. Broadly, non-clinical providers (55.0%) were more often used than clinical providers (45.0%); however, clinical providers were preferred by most women (55.6%) (Table 2). The most frequently used and preferred individual provider, among both clinical and non-clinical providers, was a private hospital, which was the first/only provider used by 25.7% of women and preferred by 31.2%. Chemists (17.3%) and pharmacies (12.8%), classified as non-clinical providers, were the next most commonly used providers and were preferred by 13.5% and 11.3% of women, respectively.

**Table 2:** Actual first/only abortion provider and preferred provider among all respondents (N = 1144)

	Actual source	Preferred source
	n (%)	n (%)
*Clinical sources*	**483 (45.0)**	**637 (55.6)**
Government hospital	85 (7.3)	155 (10.6)
Government health center	30 (2.2)	55 (3.5)
Family planning clinic	1 (0.1)	5 (0.3)
Mobile clinic (public)	1 (0.2)	1 (0.2)
Private hospital	265 (25.7)	315 (31.1)
Private doctor	98 (9.3)	103 (9.7)
Mobile clinic (private)	3 (0.3)	3 (0.3)
*Non-clinical sources*	**661 (55.0)**	**507 (44.4)**
Pharmacy	128 (12.8)	106 (11.3)
Chemist	208 (17.3)	157 (13.5)
Public event	1 (0.1)	1 (0.1)
Fieldworker (private)	8 (0.8)	6 (0.6)
Shop	53 (5.0)	38 (3.7)
Friend/relative	27 (2.4)	23 (2.0)
Healer	67 (4.1)	50 (3.5)
Market	96 (7.1)	68 (5.2)
Other	60 (4.6)	44 (3.3)
Do not know/no response	13 (0.9)	14 (1.2)

Table 3 presents reasons why women chose their first/only abortion provider/location and the primary decision-making factor, overall and by provider type (clinical, pharmacy/chemist, and other non-clinical). Convenience (41.1%), privacy/confidentiality (39.7%), location (28.9%), and cost (24.3%) were the most common reasons driving provider choice overall, but when selecting the top reason, privacy/confidentiality was the most common, reported by one-fifth of women, followed by convenience (16.9%) and recommended by someone (12.3%), most often by a friend (60.8%). The top reason varied by the type of provider. Women who first sought care from a clinical provider were significantly more likely to cite that their top reason was recommended by someone (16.1%) or that the provider had a good reputation (17.5%) compared to those who went to a pharmacy/chemist (10.0% recommended; 6.1% good reputation) or to those who sought care from other non-clinical providers (8.2% recommended; 2.6% good reputation). Those who first sought care at a pharmacy/chemist shop or relied on other non-clinical providers were significantly more likely to cite cost as their top reason (12.6% and 16.2%, respectively) compared with women who sought care from a clinical provider (5.8%). Additionally, women who sought care at a pharmacy/chemist and who indicated that their main reason was a recommendation were more likely to report that the recommender was a healthcare provider (19.5%) than those who went to clinical providers (5.5%) or to other non-clinical providers (0.0%).

**Table 3:** Factors that influenced decision to go to first/only abortion provider (N = 1144)

	Decision-making factor (multiple select)	Top decision-making factor among all respondents	Top decision-making factor among users of clinical sources (n = 483)	Top decision-making factor among users of pharmacists/chemists (n = 336)	Top decision-making factor among users of other non-clinical sources* (n = 325)	p-value
	n (%)	n (%)	n (%)	n (%)	n (%)	
Cost	305 (24.3)	128 (10.4)	**29 (5.8)**	**46 (12.6)**	**53 (16.2)**	** < 0.001**
Convenience	479 (41.1)	188 (16.9)	74 (16.0)	55 (16.8)	59 (18.5)	0.762
Location	390 (28.9)	122 (9.2)	47 (7.3)	37 (10.5)	38 (11.2)	0.212
Privacy/confidentiality	474 (39.7)	254 (20.7)	107 (18.6)	81 (23.7)	66 (20.8)	0.319
Method offered	190 (14.3)	57 (4.1)	26 (4.5)	18 (4.0)	13 (3.2)	0.686
Recommended	237 (22.3)	114 (12.3)	**62 (16.1)**	**29 (10.0)**	**23 (8.2)**	**0.017**
Partner	84 (29.3)	24 (18.6)	16 (22.9)	5 (16.9)	3 (6.1)	0.311
Family member	25 (12.2)	8 (7.8)	5 (8.2)	1 (4.7)	2 (11.2)	0.743
Friend	117 (55.1)	63 (60.8)	34 (59.8)	15 (56.8)	14 (70.1)	0.729
Health provider	21 (7.6)	9 (8.0)	4 (5.5)	5 (19.5)	0 (0.0)	0.053
Community health worker	9 (2.6)	6 (3.6)	5 (5.5)	1 (1.4)	0 (0.0)	0.324
Pharmacist	16 (1.0)	1 (0.1)	0 (0.0)	1 (0.4)	0 (0.0)	0.673
Online resources	0 (0.0)	0 (0.0)	0 (0.0)	0 (0.0)	0 (0.0)	–
Hotline (phone numbers)	0 (0.0)	0 (0.0)	0 (0.0)	0 (0.0)	0 (0.0)	–
Other	7 (2.8)	4 (2.1)	**0 (0.0)**	**0 (0.0)**	**4 (12.5)**	**0.010**
Provider had good reputation	236 (21.5)	109 (10.4)	**81 (17.5)**	**19 (6.1)**	**9 (2.6)**	** < 0.001**
Knew provider	134 (12.6)	51 (5.7)	28 (6.7)	13 (5.2)	10 (4.3)	0.594
Only option knew of nearby	99 (9.1)	49 (4.7)	13 (3.7)	19 (6.4)	17 (4.3)	0.348
Other	8 (1.1)	8 (1.1)	5 (1.9)	2 (0.4)	1 (0.4)	0.066
Do not know/no response	0 (0.0)	64 (4.7)	11 (1.8)	17 (4.2)	36 (10.4)	–

Table 4 shows the actual and preferred first/only provider/location for the subset of women who reported that they did not use their preferred provider. Of the 1144 women interviewed at follow-up, 255 (22.3%) reported that they would have preferred to go to a different provider than the first/only provider that they used. Among this group of women, 81.0% sought care from a non-clinical provider like a chemist (26.5%) or pharmacy (14.9%), and 19.0% sought care from a clinical provider; however, over three-quarters (77.4%) would have preferred a clinical provider. The largest discrepancies in actual versus preferred abortion provider/location were private hospital (7.6% actual versus 37.2% preferred), government hospital (4.3% versus 22.6%), chemist (26.5% versus 5.9%) and pharmacy (14.9% versus 6.6%). Women most often reported that cost was the reason that they did not use their preferred source (46.1%); the next most common reasons were distance from the provider (21.9%), inconvenient (16.0%), and not private (12.9%) (Table 5). Bivariate analysis of sociodemographic characteristics at the time of their abortion comparing women who did and did not use their preferred provider/location found no statistically significant differences between these two groups, except by state (Table 6).

**Table 4:** Actual versus preferred first/only abortion provider among respondents who reported that they would have preferred to use a different provider (N = 255)

	*Actual source*	*Preferred source*
	n (%)	n (%)
***Clinical sources***	**56 (19.0)**	**210 (77.4)**
Government hospital	12 (4.3)	82 (22.6)
Government health center	5 (2.6)	30 (9.7)
Mobile clinic (public)	0 (0.0)	0 (0.0)
Family planning clinic	0 (0.0)	4 (1.3)
Private hospital	26 (7.6)	76 (37.2)
Private doctor	13 (4.5)	18 (6.7)
Mobile clinic (private)	0 (0.0)	0 (0.0)
***Non-clinical sources***	**199 (81.0)**	**45 (22.6)**
Pharmacy	35 (14.9)	13 (6.6)
Chemist	66 (26.5)	15 (5.9)
Public event	0 (0.0)	0 (0.0)
Fieldworker (private)	2 (1.1)	0 (0.0)
Shop	18 (9.2)	3 (2.1)
Friend/relative	5 (2.2)	1 (0.4)
Healer	23 (7.1)	6 (3.6)
Market	31 (11.5)	3 (1.2)
Other	18 (8.3)	2 (1.1)
Do not know/No response	1 (0.1)	2 (1.8)

**Table 5:** Reasons why respondents did not use preferred provider (N = 255)

*Reasons (multiple select)*	n (%)
Cost	108 (46.1)
Inconvenient	42 (16.0)
Too far	82 (21.9)
Not private	40 (12.9)
Method not available	17 (4.1)
Provider had bad reputation	8 (1.5)
Partner encouraged use of other method	30 (10.4)
Family/friend encouraged use of other method	31 (9.1)
Provider refused	9 (3.5)
Provider not available	12 (3.1)
Side effects associated with method	22 (8.8)
Other	8 (5.2)

**Table 6:** Percent of respondents who did and did not use their preferred first/only abortion provider by sociodemographic characteristics (N = 1144)

	*Used preferred source (n* = *889)*	*Did not use preferred source (n* = *255)*	
*Characteristic*	n (row %)	n (row %)	*p-value*
Age			0.534
< 15	30 (96.2)	4 (3.8)	
15–19	167 (80.1)	48 (19.9)	
20–24	235 (81.5)	78 (18.5)	
25–29	196 (83.1)	46 (16.9)	
30–34	140 (82.9)	36 (17.1)	
35–39	71 (77.1)	24 (22.9)	
40–44	28 (78.4)	14 (21.6)	
45–49	7 (79.5)	2 (20.5)	
No response	15 (81.5)	3 (18.5)	
Married/cohabiting			0.812
No	364 (82.0)	85 (18.0)	
Yes	522 (81.4)	170 (18.6)	
Was attending school			0.341
No	663 (82.6)	192 (17.4)	
Yes	225 (79.7)	63 (20.3)	
Had children			0.329
No	412 (83.0)	97 (17.0)	
Yes	476 (80.5)	158 (19.5)	
Wealth^			0.153
Poorest	126 (79.4)	44 (20.6)	
Second poorest	187 (74.5)	73 (25.6)	
Middle	168 (84.5)	44 (15.5)	
Second wealthiest	209 (84.0)	52 (16.0)	
Wealthiest	196 (84.0)	41 (16.0)	
Residence			0.629
Rural	341 (80.8)	105 (19.2)	
Urban	548 (82.3)	150 (17.7)	
State^			**0.026**
Anambra	144 (78.6)	47 (21.4)	
Kaduna	129 (70.0)	85 (30.0)	
Lagos	202 (85.0)	36 (15.0)	
Nasarawa	138 (85.3)	27 (14.7)	
Rivers	231 (84.6)	42 (15.2)	
Taraba	45 (71.1)	18 (28.9)	

^From time of baseline survey, not time of abortion

## Discussion

Our study contributes new findings on abortion care-seeking among a general population of women in Nigeria, showing that most women in Nigeria prefer clinical sources of care. While more than half of women rely on non-clinical providers, many are able to achieve their provider preferences including accessing clinical care. These results expand on prior research which mostly relies on samples of PAC patients presenting at tertiary facilities, showing 57% of women used doctors/nurses as their abortion provider in one study compared to 8% in another that also found nearly half of women went to a non-clinical “quack” [25, 26]. We found more diversified sources of care—45% clinical providers, 30% pharmacies/chemists, and 25% other non-clinical—among a broader population of women and furthermore explored reasons and barriers to accessing preferred care.

Privacy and convenience were the top factors for choosing a provider, which aligns with qualitative research that has noted the importance of discretion and safety from social stigma to women when choosing an abortion provider [22, 23, 27, 28]. While women’s ability to operationalize their care preferences did not differ significantly by their sociodemographic characteristics, cost was an important reason why women opted for a non-clinical provider and the main barrier for not using their preferred method, followed by distance. This may point to socioeconomic inequities in women’s ability to access safe abortion methods, which is consistent with other research findings [9, 29]. These factors signal the influence of wealth and residence as contributions to disparities seen in abortion outcomes, where poor and rural women are most likely to have an unsafe abortion and least likely to seek care for complications [29].

Abortion legality in Nigeria varies by state. In Lagos state, abortion is legal to preserve the life and physical health of the woman [7, 8], compared to all other states where abortion is only legal to preserve the woman’s life. The legal environment may impact a woman’s access to clinical care by state, which represented 56.3% of abortion care in Lagos but only 30.4% in Taraba. However, these legal restrictions are more likely to inform women’s preferences rather than their ability to achieve their preferences, as we found no statistically significant differences between women who used their preferred provider/location and those who did not by state when accounting for other sociodemographic characteristics. Additional research could provide greater insight on the legal conditioning of women’s decisions in abortion care at the subnational level within Nigeria, a populous, heterogeneous country.

While harm reduction models that allow women to safely induce outside the formal healthcare system are increasingly being examined and promoted [30, 31], this analysis shows that most women seeking abortion in Nigeria would prefer to seek clinical care, such as hospitals, for safe abortion. Although provider bias and stigma are persistent issues [32, 33], women seeking abortion and PAC in other settings have reported confidence and satisfaction with services provided at upper-level clinical facilities [34]. Other factors like method preference and gestational age impact woman’s abortion care trajectories, as certain methods, like surgery, are only available at certain facility types.

Our study has some limitations, including the risk of differential underreporting of abortion by provider/location, which would bias our observed findings. However, we do not think this bias would qualitatively change our overall conclusions that a significant proportion of women prefer a clinical provider but seek care from a non-clinical provider due to financial constraints. Another limitation is the potential misclassification of abortion provider/location, as some women are reporting their abortion experience several years after it took place and could be conflating different abortion events. While focusing on personal preference and decision-making factors, this analysis does not explore all the reasons why a woman may opt to go to a certain provider, such as gestational age. In addition, women go through different pathways to obtain an abortion, not all of which are complete with one visit to one particular provider.

This study also has a number of key strengths. The use of a large, population-based survey and the follow-up survey allowed for more detailed questions about the woman’s abortion experience from a large sample of women. The use of a population-based survey also allowed us to capture the population of women seeking abortion care from both formal and informal healthcare settings, which provides a more representative range of abortion experiences and preferences in Nigeria. The findings provide important insight regarding factors that would need to be addressed to better meet women’s abortion care needs.

## Conclusions

These findings add to the literature on women’s abortion trajectories, associated decision-making, and barriers to care, highlighting the diversity of experiences and provider preferences, and the need for an expansion of legal, low-cost abortion services in clinical healthcare settings to better meet women’s reproductive healthcare needs. In the meantime, harm reduction efforts should seek to increase awareness of medication abortion drugs to reduce women’s reliance on non-recommended abortion methods, regardless of provider. Additionally, continued expansion of voluntary family planning services to prevent unintended pregnancy and increased availability of quality PAC services can help decrease unsafe abortion related morbidity and mortality.

## Data Availability

The dataset for the 2019–2020 follow-up survey is available from the corresponding author on reasonable request. The dataset for the 2018 baseline survey can be accessed by submitting a request through the PMA website: https://datalab.pmadata.org/.

## Abbreviations

CRERD: Centre for Research, Evaluation Resources and Development
PAC: Post abortion care
PMA: Performance Monitoring for Action
